# Biomarker role of maternal soluble human leukocyte antigen G in pre‐eclampsia: A meta‐analysis

**DOI:** 10.1002/iid3.1254

**Published:** 2024-04-19

**Authors:** Abhinav Bhattarai, Sangam Shah, Krishna Dahal, Raksha Neupane, Sangharsha Thapa, Niraj Neupane, Joshuan J. Barboza, Anisha Shrestha, Ranjit Sah, Vasso Apostolopoulos

**Affiliations:** ^1^ Institute of Medicine Tribhuvan University Maharajgunj Nepal; ^2^ Department of Neurology Westchester Medical Center Valhalla New York USA; ^3^ Rochester General Hospital Rochester New York USA; ^4^ Vicerrectorado de Investigacion Universidad Norbert Wiener Lima Peru; ^5^ Department of Microbiology Tribhuvan University Teaching Hospital, Institute of Medicine Kathmandu Nepal; ^6^ Department of Microbiology Dr. D. Y. Patil Medical College, Hospital and Research Centre, Dr. D. Y. Patil Vidyapeeth Pune India; ^7^ Department of Public Health Dentistry Dr. D.Y. Patil Dental College and Hospital, Dr. D.Y. Patil Vidyapeeth Pune India; ^8^ Institute for Health and Sport, Immunology and Translational Research Victoria University Melbourne Victoria Australia; ^9^ Australian Institute for Musculoskeletal Science, Immunology Program Melbourne Victoria Australia

**Keywords:** biomarker, human leukocyte antigen G, pre‐eclampsia, sHLA‐G

## Abstract

**Introduction:**

Human leukocyte antigen‐G (HLA‐G) is a non‐classical class I HLA molecule shown to regulate the immunomodulation of maternal immune cells to prevent fetal tissue destruction. Low levels of freely circulating maternal soluble HLA‐G (sHLA‐G) have been observed in pre‐eclampsia, however, no pooled evidence exists. This meta‐analysis aimed to generate pooled findings on the association of sHLA‐G levels with pre‐eclampsia and is the first study to perform a trimester‐wise comparison of the levels of sHLA‐G in preeclamptic cases and normal pregnant controls.

**Methods:**

The databases PubMed, Emba, Web of Science, and Google Scholar through May 31, 2023. Preeclamptic women were defined as cases and normal pregnancies as controls. Data on the level of sHLA‐G in cases and controls was extracted and subjected to a meta‐analysis using a random‐effects model. The pooled effect was expressed in terms of standardized mean difference (SMD). Sensitivity analysis was performed to investigate the effect of the exclusion of each study on the pooled results. Publication bias was assessed statistically.

**Results:**

Nine studies with altogether 567 PE cases and 1132 normal pregnancy controls were included in the meta‐analysis. The first and third trimester levels of sHLA‐G in PE cases were significantly lower than that of normal pregnant controls: (SMD: −0.84 [−1.29; −0.38]; *p* = .003; I^2^ = 54%) and (SMD: −0.39 [−0.71; −0.06]; *p* = .02; I^2^ = 79%) respectively. Sensitivity analysis revealed significant fluctuations in the pooled findings when few studies were excluded, raising questions on the consistency of results among studies.

**Conclusion:**

Although we found that first and third‐trimester sHLA‐G levels in pre‐eclampsia are significantly lower, taking into consideration the inconsistent results from the sensitivity analysis, our findings advocate the demand for more studies with larger sample sizes to generate solid ground pooled evidence on the predictive role of sHLA‐G in pre‐eclampsia.

## 1 INTRODUCTION

Generally, the immune system functions to destroy foreign antigens, which is not the case in pregnancy. Pregnancy is a distinctive physiological event in which a semi‐allogeneic fetus survives in the uterus of the mother without being rejected, despite significant exchange in the circulation. The molecular mechanisms for the survival of the fetus in an antigenically different mother is enigmatic.^1^ This process has been linked to immune tolerance via which maternal antibodies by fetal alloantigens are suppressed.^2^ This suppressive mechanism takes place on the endometrial decidua, the site where the placental bed is established. The endometrial decidua is richly supplied by spiral arteries whose endothelium is eventually invaded by extra villous trophoblasts differentiated from the fetus.^3^ The invasion occurs in two stages, at 8–10 weeks and then at 16–18 weeks of gestation.^4^

**Figure 1:**
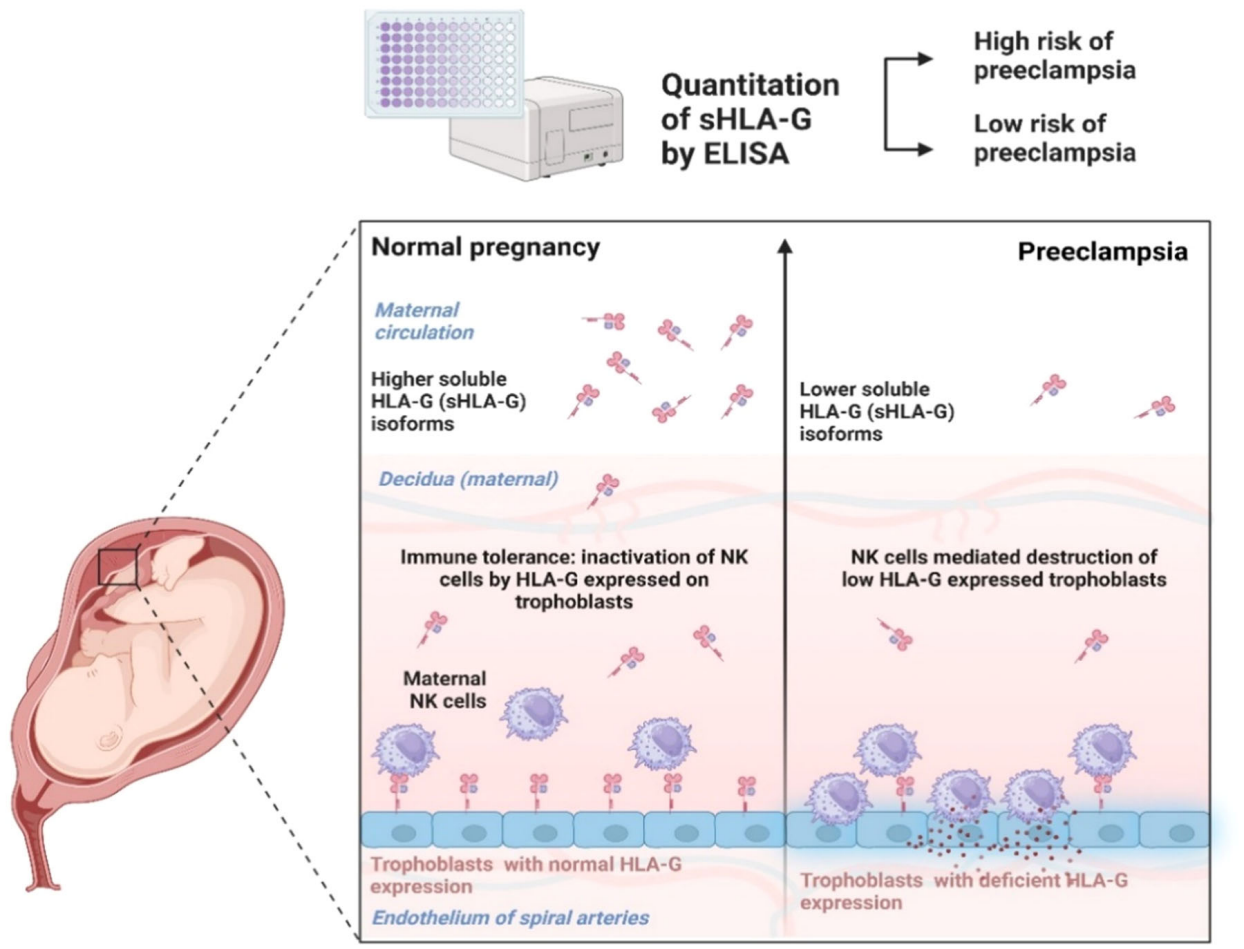
Pregnancy outcome based on HLA‐G expression on trophoblasts and utility of soluble isoforms.

Human leukocyte antigen‐G (HLA‐G) is a nonclassical class I HLA molecule shown to regulate the immunomodulation of maternal immune cells in the decidua to prevent fetal tissue destruction.^5^ HLA‐G is expressed by extra villous trophoblasts in the spiral artery and interstitium and is translated as seven distinct isoforms: HLA‐G1, ‐G2, ‐G3, and ‐G4 as membrane‐bound, and HLA‐G5, ‐G6, and ‐G7 as secreted soluble forms (sHLA‐Gs). Majority of the secreted forms is HLA‐G5 and a significant portion of the membrane bound HLA‐G1 is proteolytically cleaved in free soluble form which forms a dimer with sHLA‐G5 and circulate in the maternal circulation.^6^, ^7^ HLA‐G1/G5 have been extensively studied, structurally, molecularly, and are used in commercial enzyme‐linked immunosorbent assay (ELISA) kits for their detection.^8^ HLA‐G is a major regulator for fetal survival, and, achieves a successful pregnancy via three mechanisms, spiral artery remodeling, facilitation of fetal growth, and immune tolerance.^3^ The endometrial decidua is rich in natural killer (NK) cells and any encounter with fetal alloantigens can cause immune activation and killing. However, this is inhibited by trophoblastic HLA‐G receptor interaction and protects fetal NK‐sensitive cells from destruction (Figure 1).^9^ Evidence of the protective function of HLA‐G to trophoblastic cells has been established by experimental studies where no cytotoxic reactions were observed between maternal NK cells and extra villous trophoblasts in cell cultures. However, cytotoxic effects were indeed observed when anti‐HLA‐G antibodies were used to block the expression, and these findings implicate the certainty of HLA‐G's security on trophoblasts.^10^ In addition, low expression profiles of HLA‐G correlate with complications in pregnancy, including pre‐eclampsia.

Pre‐eclampsia has a worldwide incidence of 2%–8% of pregnancies and is the top‐tier red flag for risk of maternal and fetal mortality.^11^ Immune tolerance failure is an important cause of pre‐eclampsia.^12^ Deficient states of HLA‐G have been implicated in the progression of pre‐eclampsia via poor spiral artery remodeling, and failure to prevent maternal immune activation against fetal alloantigens.^13^ The condition is diagnostically defined by the American College of Obstetricians and Gynecologists (ACOG) as systolic and diastolic blood pressure greater than 140 and 90 mm of Hg respectively, detected twice within 4 h and occurring after 20 weeks of pregnancy in a previously normotensive woman.^14^ Additionally, the criteria have been supplemented with a 24‐h protein >300 mg or protein‐to‐creatinine ratio >0.3 mg or urine dipstick reading of 2+ on the albumin label.^15^ Additionally, in the absence of proteinuria, the presence of new onset hypertension, one of the following: thrombocytopenia (platelet count <100 *10^9^/L), renal insufficiency (serum creatinine >1.1 mg/dL), impaired liver function (liver enzymes raised to twice the normal concentration), pulmonary edema, or new‐onset medication‐unresponsive headache is diagnostic of pre‐eclampsia.^14^ Considering the role of HLA‐G in immune tolerance, studies in the last two decades suggest its significant associations with pre‐eclampsia.

Using immunohistochemistry and in situ hybridization studies, low HLA‐G expression has been noted in preeclamptic women.^16^, ^17^ Genetic polymorphism studies in the HLA‐G gene have shown similar findings with 14 base pair insertion/deletion polymorphism.^18^ Similarly, sHLA‐G in maternal circulation are lower in preeclamptic women,^19^, ^20^, ^21^, ^22^ although, some studies have shown no significant difference as compared to normal pregnant controls,^23^, ^24^, ^25^, ^26^ raising the question of whether the estimation of maternal blood sHLA‐G actually aids in classifying pregnant subjects at risk of pre‐eclampsia.

From a routine diagnostic point of view, sHLA‐G can be easily identified as compared to the invasive procedures of immunohistochemistry and molecular techniques. Although pre‐eclampsia has been extensively studied, it is still diagnosed at onset and there lies a crucial demand for appropriate biomarkers that allow foreseeing the diagnosis. sHLA‐G is one such promising biomarker, and currently, there is a need for an appropriate meta‐analysis to be conducted to generate pooled findings on the association of maternal sHLA‐G levels with the occurrence of pre‐eclampsia. This will aid gynecologists and obstetricians to distinguish pregnant women at risk of pre‐eclampsia. As such, the current meta‐analysis herein, aimed to generate pooled findings on the association between sHLA‐G levels with pre‐eclampsia. This is the first meta‐analysis to perform a trimester‐wise comparison of the levels of sHLA‐G in preeclamptic cases and normal pregnant controls.

## 2 METHODS

### 2.1 Protocol and registration

This is a systematic review with meta‐analysis performed in accordance with the PRISMA (Preferred Reporting Items for Systematic Reviews and Meta‐analysis) guidelines^27^ and is registered in the International Prospective Register of Systematic Reviews (PROSPERO) as CRD42023430544.

### 2.2 Data sources and search strategy

The databases PubMed, Embase, Web of Science, and Google Scholar were comprehensively searched for relevant literature published from the inception of these databases until the May 31, 2023. The search terms ‘pregnancy’, ‘complication’, ‘pre‐eclampsia’, ‘human leukocyte antigen G’, ‘soluble human leukocyte antigen G’, ‘HLA‐G’, and ‘sHLA‐G’, were connected by ‘AND’ and ‘OR’ Boolean operators to formulate a search string.

### 2.3 Study selection

The study selection was performed in multiple steps by authors AB and RN. First, the results retrieved from each database were imported into Mendeley Desktop version 1.19.8 and scanned for duplicates. The duplicates were removed automatically. The title and abstracts were screened, and studies were sorted. Full‐text of the sorted studies were retrieved and selected based on the eligibility criteria. The references of the selected studies were also screened to obtain relevant results. For studies whose full text were not available, the corresponding authors were emailed. All discrepancies were discussed and resolved by a third reviewer, author ST.

### 2.4 Eligibility criteria

All studies that possessed the following characteristics were considered eligible for inclusion in the meta‐analysis.

1. Preeclamptic women as cases: pre‐eclampsia defined as systolic blood pressure/diastolic blood pressure >140/90 mmHg and proteinuria >300 mg/24 h; protein: creatinine >0.3 mg, or urine albumin 2+ on the dipstick.
2. Normal pregnancies as controls.
3. sHLA‐G levels estimated in accordance with trimester.

Review articles, case reports, editorials, abstract presentations, book chapters, case series, and articles in non‐English languages were subjected to exclusion.

### 2.5 Risk of bias assessment

The risk of bias was assessed using the Newcastle‐Ottawa Scale (NOS).^28^ The scale consists of 3 domains^1^ Selection,^2^ Comparability, and^3^ Outcome where the study is judged and scores are assigned, 9 being the highest. Studies with scores ranging from 7 to 9, 4 to 6, and 0 to 3 were considered low‐risk, high risk and very high‐risk respectively. The checklist for the score assignment in each domain for the study are included in the supplementary material. Briefly, the studies were carefully checked for case definition, biomarker measurement, control assignment, and outcome.

### 2.6 Data extraction and synthesis

Data extraction was performed on a pre‐specified data extraction sheet in Microsoft Excel®. Data relating to the following categories were extracted^1^: author and year,^2^ study country,^3^ number of PE cases,^4^ number of controls,^5^ Age of PE subjects,^6^ trimesters of sHLA‐G estimations,^7^ Gestational age at sampling,^8^ gravidity,^9^ estimation methodology,^10^ sHLA‐G levels in PE cases, and^11^ sHLA‐G levels in controls. Continuous data were reported as mean (standard deviation) or median (interquartile range). Specifically, in the case of sHLA‐G levels, data expression varied. While few studies reported levels as mean (SD), others reported the levels as median (IQR). For achieving data compatibility to perform a meta‐analysis, all data on the levels of sHLA‐G expressed as median (IQR) were converted into mean (SD) as per Hozo et al.'s guideline.^29^

### 2.7 Statistical analysis

The statistical analyses were performed on MedCalc® Statistical Software. The levels of sHLA‐G of PE cases and normal pregnant controls were pooled in accordance with different trimesters. Considering the differences in the expression units (U/mL, ng/mL, etc.) and to adjust the variability present in each study, the standardized mean difference (SMD) was used to express the pooled difference in the sHLA‐G levels between cases and controls.^30^ A random‐effects model was used to express the pooled findings. The heterogeneity was estimated using the*I* ^*2*^ statistics. Results of *I* ^*2*^ ranging 0%–40%, 30%–60%, 50%–90%, and 75%–100% were considered as low, moderate, substantial, and considerable heterogeneity respectively.^31^ Inter‐study heterogeneity was expressed in terms of *tau* ^*2*^ as obtained by the DerSimonian and Laird method. Forest plots were generated for interpretation. The difference in the sHLA‐G levels between cases and controls was considered statistically significant when the *p‐*value was <.05. To analyze the consistency in the results in each included study and to investigate the effect of the exclusion of each study on the pooled findings, sensitivity analysis was performed by the exclusion of studies one at a time. The publication bias was assessed statistically by Egger's and Begg's test, and visually by funnel plots.

## 3 RESULTS

### 3.1 Search results and study selection

**Figure 2:**
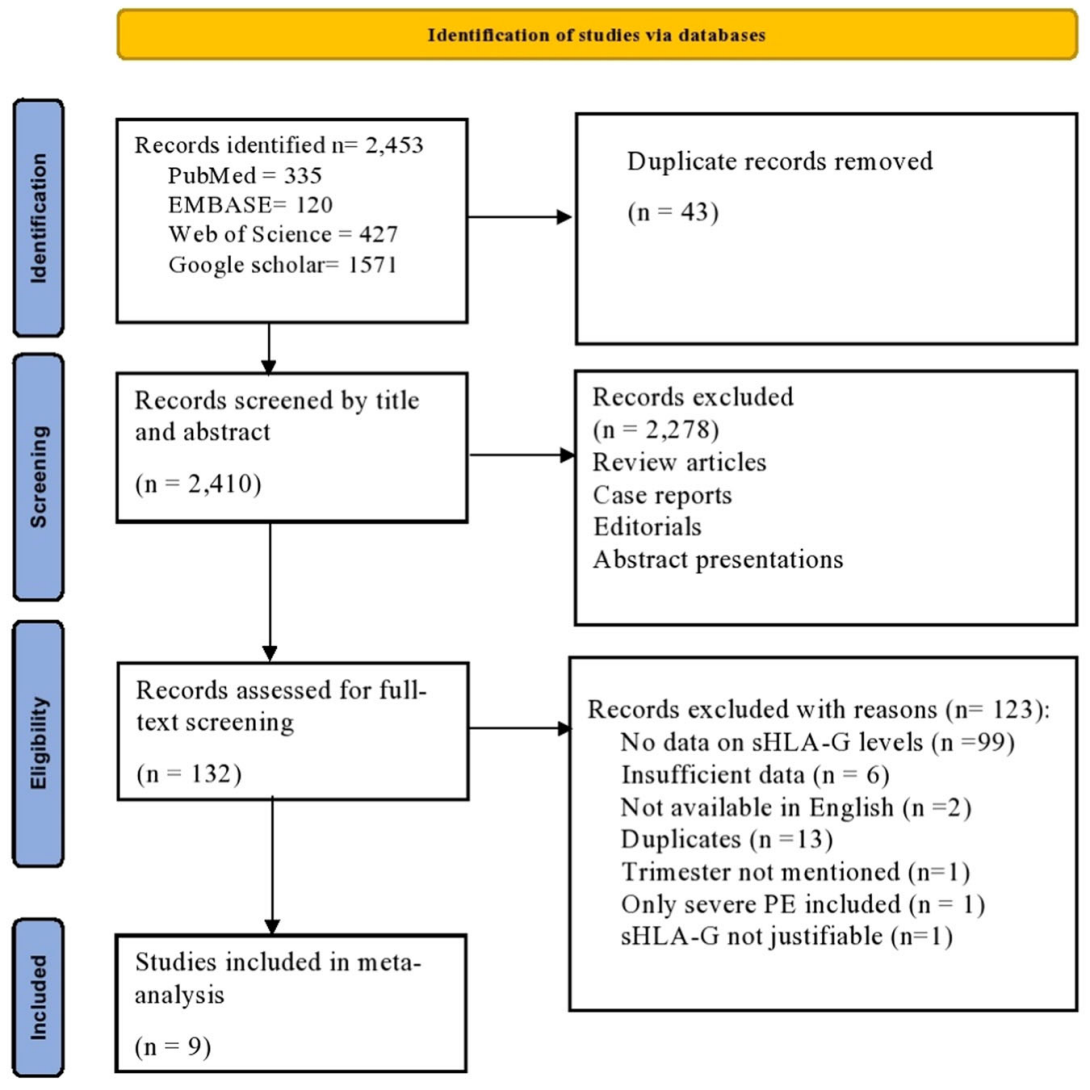
Prisma guidelines.

The preliminary search results retrieved 2453 records which were screened for duplicates followed by title and abstract screening. 132 articles were subjected to full‐text screening based on the eligibility criteria and 9 studies^19^, ^20^, ^21^, ^22^, ^23^, ^24^, ^25^, ^26^, ^32^ were included in the meta‐analysis. The study selection process is displayed in the PRISMA flowchart (Figure 2).

### 3.2 Risk of bias assessment

The results of the risk of bias assessment are shown in the supplementary table. Briefly, all studies except Steinborn et al. 2007^32^ possessed a low risk of bias (score 7–9). We perceived that this study had a high risk of bias (score 6) since the demographic status of PE cases and controls including age, gravidity, and clinical data were not reported. Based on the risk of bias scores, no studies were subjected to exclusion from the analysis.

### 3.3 Descriptive characteristics of the included studies

Nine studies with altogether 567 PE cases and 1132 normal pregnancy controls were included. Except for Yie et al.,^22^ all studies classified the population into PE based on ACOG's criteria. Although the criterion followed in Yie et al.'s study wasChesley's,^33^ it was included in the analysis since the biochemical evaluation for PE was elucidated to be identical. Except for 3 studies (Kolarz et al., Marozio et al., and Steinborn 2007 et al.), all studies were prospective. The age of PE cases ranged between 20 and 40 years old. The sHLA‐G levels in both PE cases and controls were reported in all trimesters in the study of Yie et al., whereas others reported that in either any one or two trimesters. Overall, the data on sHLA‐G levels between PE cases and controls in the first, second, and third trimesters were assessable from three, three, and seven studies respectively. All pregnancies were singleton. The gestational age at sampling in all trimesters was similar. sHLA‐G levels in all studies were estimated by ELISA assays. The findings on the association of sHLA‐G levels with PE varied among the studies, which suggests the need to generate pooled findings. The descriptive characteristics of the included studies are highlighted in Table 1.

**Table 1:** Descriptive characteristics of the included studies.

Author and year (*Citations*)	Study country	No. of participants		Age		Trimesters of sHLA‐G estimation	Gestational age at sampling		Gravidity		Parity		sHLA‐G estimation method	Findings	NOS score
		PE	Controls	PE	Controls		PE	Controls	PE	Controls	PE	Controls			
Biyik et al. 2014^23^	Turkey	19	154	28.7 ± 6.6	27.24 ± 6.21	1^st^	87.42 ± 5.54 *days*	86.65 ± 5.75 *days*	2.47 ± 1.50	2.44 ± 1.56	1.00 (0–4)	1.06 ± 1.20	ELISA	*A*; did not predict risk of PE	9
						2^nd^	NR	NR							
Garcia et al. 2018^24^	Mexico	8	8	27.4 ± 7.2	22.3 ± 3.3	3^rd^	37.4 ± 2.8 *weeks*	38.9 ± 1.5 *weeks*	1.82 ± 1	1.86 ± 1.1	NR	NR	ELISA	*A*	8
Jacobsen et al. 2020^19^	Norway	139	152	32.3 (29.8‐35.3)	37.7 (31.5‐36.4)	3^rd^	36.4 (39.4 −37.9) *weeks*	39.0 (38.7–39.4) *weeks*	NR	NR	NR	NR	ELISA	*B*	8
Kolarz et al. 2012^20^	Poland	35	52	28.43 ± 4.67	28.07 ± 4.83	3^rd^	33.02 ± 3.45 *weeks*	33.97 ± 3.48 *weeks*	1.79 ± 1.66	1.72 ± 0.91	1.56 ± 0.83	1.39 ± 0.69	ELISA	*B*	9
Marozio et al. 2017^25^	Italy	65	234	31.7 ± 4.8	31.7 ± 4.9	1^st^	NR	NR	NR	NR	NR	NR	ELISA	*A*	7
Rokhafrooz et al. 2018^21^	Iran	150	150	30.31 ± 3.65	30.25 ± 3.58	3^rd^	35.51 ± 1.97 *weeks*	36.01 ± 1.75 *weeks*	3.4 ± 1.5	3.5 ± 1.7	NR	NR	ELISA	*B*	9
Steinborn et al. 2003^26^	Germany	55	145	NR	NR	2^nd^, 3^rd^	NR	NR	NR	NR	NR	NR	ELISA	2^nd^: *A* 3^rd^: *A*	7
Steinborn et al. 2007^32^	Germany	84	291	NR	NR	2^nd^, 3^rd^	NR	NR	NR	NR	NR	NR	ELISA	2^nd^: *B* 3^rd^: *A*	6
Yie et al. 2005^22^	USA	12	12	28.0 ± 5	29.8 ± 2.2	1^st^	8.2 ± 0.6	7.8 ± 0.5	NR	NR	NR	NR	ELISA	1^st^: *B* 2^nd^: *B* 3^rd^: *A*	8
						2^nd^	19.3 ± 1.3	17.8 ± 0.8							
						3^rd^	37.3 ± 1.3	37.8 ± 1.4							

Abbreviations: A, no statistically significant difference in the levels of sHLA‐G between PE cases and controls; B, significantly lower sHLA‐G levels in PE cases than controls; ELISA, enzyme‐linked immunosorbent assay; NOS, Newcastle‐Ottawa Scale; NR, not reported; PE, Preeclampsia;pre‐eclampsia.

### 3.4 Meta‐analysis on the sHLA‐G levels between PE cases and controls

#### 3.4.1 First trimester

**Figure 3:**
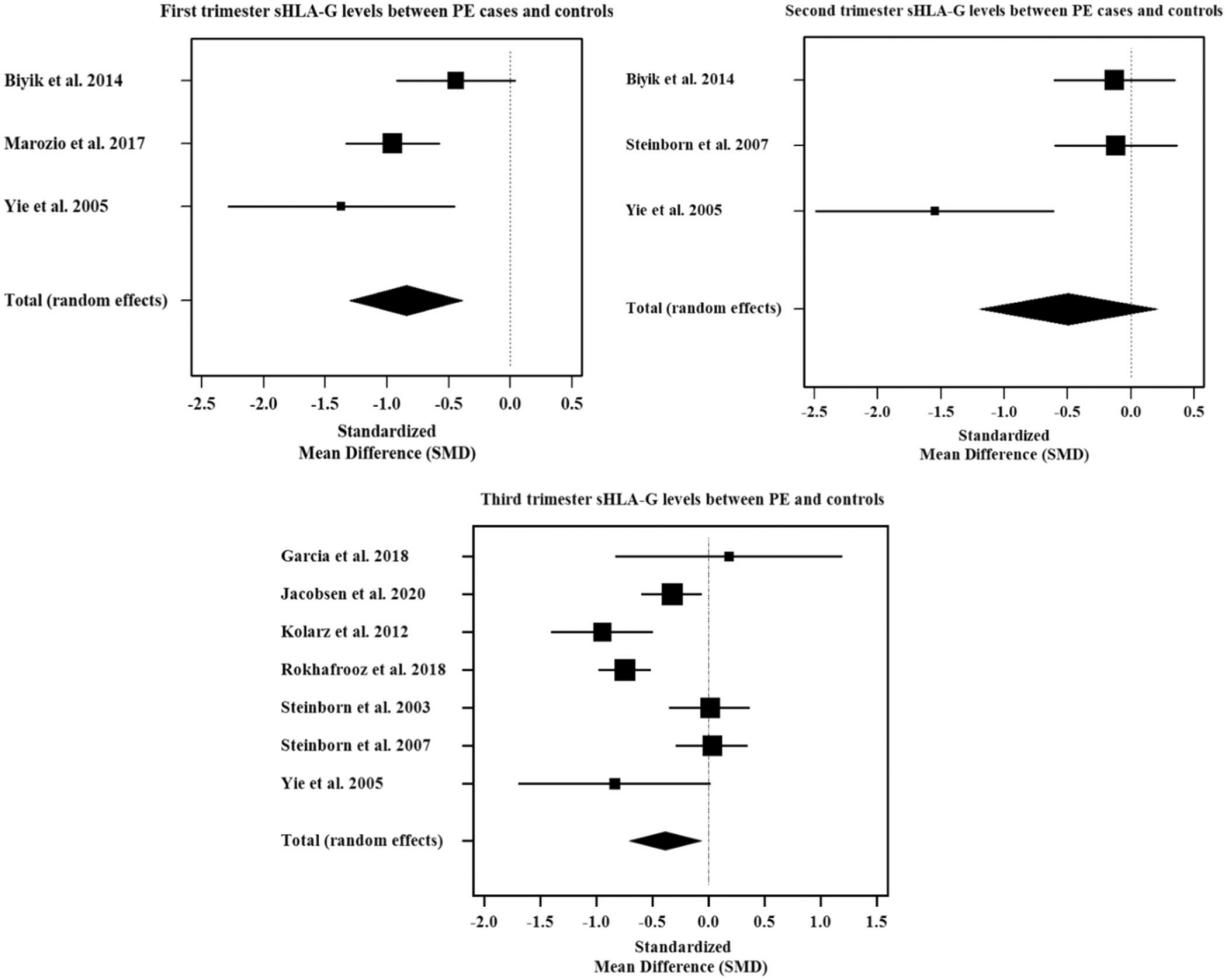
Forest plot showing the trimester wise sHLA‐G levels between PE cases and controls.

**Figure 4:**
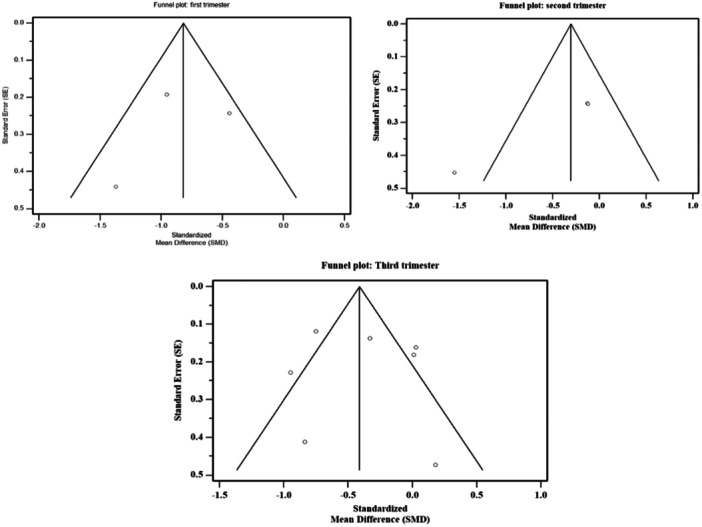
Funnel plots of studies investigating sHLA‐G levels in pre‐eclampsia cases and controls.

Three studies reported the first‐trimester sHLA‐G levels in 63 PE cases and 400 controls. The heterogeneity was substantial. The first‐trimester levels of sHLA‐G in PE cases were significantly lower than that of normal pregnant controls [SMD: −0.84 (−1.29; −0.38); *p* = .003; *I* ^2^ = 54%]. The forest plot (Figure 3) shows the pooled findings on the first trimester sHLA‐G levels between PE cases and controls. Sensitivity analysis revealed that this significant finding changed to insignificant when one study was removed from the analysis (Table 2). There were no significant publications bias detected [*p*‐value (Egger's test = .7766) and (Begg's test = .6015)]. Funnel plot (Figure 4) shows no outliers, clarifying the absence of publication bias.

**Table 2:** Sensitivity analysis showing the changes in the results when studies were excluded.

Study excluded	Resultant Pooled SMD	Resultant *p* value
**First trimester**		
Biyik et al.	−1.02 [−1.36; −0.67]	<.00001
Marozio et al.	−0.82 [−1.72; 0.07]	**.07** *
Yie et al.	−0.72 [−1.22; −0.22]	.005
**Second trimester**		
Biyik et al.	−0.78 [−2.17; 0.62]	.28
Steinborn et al.	−0.78 [−2.17; 0.61]	.27
Yei et al.	−0.12 [−0.46; 0.21]	.47
**Third trimester**		
Garcia et al.	−0.43 [−0.77; −0.09]	.01
Jacobsen et al.	−0.40 [−0.81; 0.01]	**.06** *
Kolarz et al.	−0.29 [−0.63;0.04]	**.09** *
Rokhafrooz et al.	−0.30 [−0.63;0.03]	**.07** *
Steinborn et al. 2003	−0.46 [−0.81; −0.12]	.009
Steinborn et al. 2007	−0.47 [−0.81; −0.14]	.006
Yie et al.	−0.34 [−0.69; −0.00]	.05

* Insignificant.

#### 3.4.2 Second trimester

Three studies reported the second‐trimester sHLA‐G levels in 50 PE cases and 296 controls. Considerable heterogeneity was present. The second‐trimester levels of sHLA‐G in PE cases were lower than that of normal pregnant controls, however, it was statistically insignificant [SMD: −0.48 (−1.16; −0.20); *p* = .17; *I* ^2^ = 75%]. The forest plot (Figure 3) shows the pooled findings on the second trimester sHLA‐G levels between PE cases and controls. Sensitivity analysis showed no fluctuation in the findings when each study was excluded from the analysis (Table 2). Funnel plot (Figure 4) shows an outlier, indicating the presence of publication bias which was statistically significant as detected from the Egger's test. *(p*‐value [Egger's test = .0166] and [Begg's test = .6015]).

#### 3.4.3 Third trimester

Seven studies reported the third‐trimester sHLA‐G levels in 406 PE cases and 544 normal pregnant controls. Considerable heterogeneity was present. The third‐trimester levels of sHLA‐G in PE cases were significantly lower than that of normal pregnant controls [SMD: −0.39 (−0.71; −0.06); *p* = .02; *I* ^2^ = 79%]. The forest plot (Figure 3) shows the pooled findings on the third trimester sHLA‐G levels between PE cases and controls. Sensitivity analysis revealed that this significant finding changed to insignificant when three studies were removed from the analysis (Table 2). Although the funnel plot (Figure 4) showed few outliers, there were no significant publication bias detected *(p*‐value [Egger's test = .7972] and [Begg's test = .6523]).

## 4 DISCUSSION

Pre‐eclampsia being a potentially fatal complication, can severely influence maternal and fetal outcomes in pregnancy.^34^ Despite being a very common obstetric complication, the condition is diagnosed on onset and currently there lies a need of a predictive biomarker that can aid in foreseeing the risk of pre‐eclampsia in pregnant women. Although several markers are being investigated, there is barely any FDA‐approved predictive tests for pre‐eclampsia.^35^, ^36^ In May 2023, the FDA approved a commercial blood spot testing kit created by Thermo Fisher Scientific. This kit relies on the ratio of placental proteins, specifically placental growth factor and soluble fms‐like tyrosine kinase‐1. It marks the first predictive test for pre‐eclampsia. However, its actual impact on predicting and managing pre‐eclampsia in clinical settings remains uncertain. And certainly, there lies the need of research and development of such additional predictive tests for foreseeing the risk of pre‐eclampsia and imparting timely prophylaxis.^37^

Amidst the discovery and investigations on different markers for predicting pre‐eclampsia, sHLA‐G is one such aspiring marker that has been extensively studied. The molecule is known to protect against pre‐eclampsia via the remodeling of spiral arteries, immunological tolerance on maternal immune cells, and fetal development. There have been extensive studies on the association of genetic polymorphisms in the HLA‐G gene and HLA‐G placental expression with the occurrence of pre‐eclampsia and significant pooled findings have been reported by a meta‐analysis indicating higher susceptibility in the presence of a 14 base pair insertion/deletion polymorphism.^18^ On the other hand, there exists no solid ground pooled evidence on the level of soluble isoforms of HLA‐G and the occurrence of pre‐eclampsia. In this study, we evaluated the findings of several. studies which compared sHLA‐G levels between preeclamptic cases and normal pregnant controls to generate a pooled finding.

The analysis revealed low sHLA‐G levels in preeclamptic women as compared to women with normal uncomplicated pregnancies. However, the findings were statistically significant only in the first and third trimesters. Insignificant differences in the sHLA‐G levels between cases and controls were noted in the second trimester. Importantly, the sensitivity analysis revealed frequent fluctuations of the pooled findings and changes in the significance when studies were subjected to exclusion. In the first trimester, the exclusion of one study led to significant deviation in the pooled findings while in the case of the third trimester, there were a few more studies whose exclusion affected the findings. These observations imply that our pooled findings, although significant in the first and third trimesters, have been sensitive to variations in individual study findings and it raises a question about the consistency of the universal results on the association of sHLA‐G levels with pre‐eclampsia. Hadn't the pooled findings fluctuated significantly upon the exclusion of individual studies, then only the robustness of the association could be guaranteed. These could be due to differences in the study sample sizes, study design, diversity of pre‐eclampsia cases (early‐onset and late onset), varying severities (proportion of participants with mild/severe pre‐eclampsia), or ethnic variations of HLA‐G transcriptome.^38^

Although sHLA‐G levels were significantly lower in the first and third trimesters in preeclamptic women according to the analysis, considering the fluctuations in the sensitivity analysis and considerable heterogeneity, we do not state the existence of a predictive role confidently. However, one must note that the study which led to insignificance in the pooled findings upon exclusion was indeed influential. This can be justified by the relatively large sample size of Marozio et al.^25^ which when excluded led to insignificant results. Likewise, other studies^20^, ^21^, ^26^, ^32^, ^39^ whose exclusion led to insignificant findings in the third trimester too had a comparatively larger sample size as compared to other studies whose exclusion didn't fluctuate the pooled finding. Therefore, there lies the need for more studies with a larger population so that a more robust result can be generated, and a solid ground pooled finding can be assured. Interestingly, there was no fluctuation in the sensitivity analysis in the second trimester, *which we assume to be a play in the sensitivity analysis by the relative sample sizes of the included studies, instead of an actual process during the second trimester. After all studies have shown consistent trajectory of sHLA‐G levels with progress in gestational age with no skews in between*.^40^

We did notice that there were only a few studies reporting sHLA‐G levels in the first and second trimesters whereas, there were a higher number of studies reporting the biomarker levels in the third trimester. Most studies have emphasized the comparison of sHLA‐G levels between pre‐eclampsia cases and controls in the late trimester. However, from a diagnostic point of view, it is undoubtedly rational to establish the performance of a predictive biomarker in the early stage rather than during the stage of occurrence. Taking into attention that a majority of cases of pre‐eclampsia are detected in the late second and the third trimesters,^41^, ^42^ biomarker that possesses better predictive value in the first trimester would undoubtedly be clinically advantageous than having that in the second or third trimester where the onset of pre‐eclampsia is vulnerable and prophylaxis at this stage might prove unfavorable.

Additionally, since higher sHLA‐G levels are present in the first trimester and the levels decline with gestational progress,^40^ from an assay detection and linearity perspective, it is favorable to emphasize sHLA‐G levels in the first trimester. A recent meta‐analysis found that the first‐trimester sHLA‐G in preeclamptic women was significantly lower than that in normal pregnant controls.^43^ Unfortunately, an in‐depth appraisal of this meta‐analysis could not be performed due to irretrievable full‐text, and we received no response when the authors were emailed. Nevertheless, it seems that first‐trimester sHLA‐G level does possess a predictive utility in pre‐eclampsia unless results from more studies with larger participants are obtained.

Adhering to the utility of sHLA‐G in predicting pre‐eclampsia, although the findings from our meta‐analysis have not been very promising, sHLA‐G appears to be associated with successful pregnancy outcomes. Apart from pre‐eclampsia, lower sHLA‐g levels have also been implicated in other pregnancy complications including placental abruption, rupture of membrane, intrauterine growth retardation, preterm delivery, and recurrent spontaneous abortions.^44^, ^45^, ^46^, ^47^ Furthermore, sHLA‐G has also been implicated in successful implantation and pregnancy outcome following in vitro fertilization (IVF). Radwan et al.^48^ investigated the sHLA‐G levels in embryo cultures that were implanted for pregnancy and found that embryo cultures that led to pregnancy and live births had a higher sHLA‐G level in the culture medium. Likewise, the selection of fertilized oocytes with the highest HLA‐G expression was found to produce a higher rate of clinical pregnancies in IVF. These findings further corroborate the certainty of a favorable activity of HLA‐G on pregnancy.

Our study is the first to our knowledge, that performed a pooled comparison on the levels of sHLA‐G between preeclamptic women and normal pregnant women in accordance with all three trimesters. We have stringently adhered to the criteria of pre‐eclampsia while inspecting the patient selection within the study which aided in a homogeneous comparison and rational data pooling. As per the quality control step in a meta‐analysis, we have emphasized and carefully interpreted our findings in accordance with the results from our sensitivity analysis. However, our study holds certain limitations. To achieve a homogeneous data pooling, we excluded a few studies with relevant data on sHLA‐G that could not be incorporated into the meta‐analysis.^49^, ^50^, ^51^, ^52^ The sample sizes of our included studies were varied, overall, the sizes were small, considerable heterogeneity was detected and significant fluctuations were noted in the sensitivity analysis which prevented us from confidently aligning our findings to the assumed predictive utility of sHLA‐G in pre‐eclampsia.

## 5 CONCLUSION

Our pooled findings revealed significantly lower maternal sHLA‐G levels in preeclamptic women as compared to normal pregnant women in the first and third trimesters. In the second trimester, although the sHLA‐G was lower in preeclamptic women, it was statistically insignificant. We noted that the exclusion of few studies affected the pooled findings which indicates inconsistencies in the sample size and results, obscuring the assumed predictive role of the biomarker. Our findings therefore highly advocate the demand for further studies with larger sample sizes to aid generate solid ground pooled evidence to uncover the predictive role of sHLA‐G in pregnant women with pre‐eclampsia which will be a foundation for the performance of large‐scale validation studies to determine the applicability of the biomarker in routine clinical practice.

## AUTHOR CONTRIBUTIONS

**Abhinav Bhattarai**: Conceptualization; data curation; project administration; visualization; writing—original draft. **Sangam Shah**: Formal analysis; investigation; software; writing—original draft. **Krishna Dahal**: Resources; visualization; writing—original draft. **Raksha Neupane**: Writing—original draft. **Sangharsha Thapa**: Writing—review and editing. **Niraj Neupane**: Writing—review and editing. **Joshuan J. Barboza**: Software. **Anisha Shrestha**: Writing—review and editing. **Ranjit Sah**: Supervision; Writing—review and editing. **Vasso Apostolopoulos**: Conceptualization; supervision; finalization.

## CONFLICT OF INTEREST STATEMENT

The authors declare that they have no conflicts of interest.

## Supporting information

Supporting information.

## Data Availability

The required data can be accessed from the corresponding author.
