# Supplementary material for: Biomarker role of maternal soluble human leukocyte antigen G in pre‐eclampsia: A meta‐analysis
Source: Immun Inflamm Dis. 2024 Apr 19;12(4):e1254. doi: 10.1002/iid3.1254 (PMC11027746; doi:10.1002/iid3.1254)
Supplement: Supplementary file 1 — Supporting information. [file IID3-12-e1254-s001.docx]

**Supplementary material**

Newcastle-Ottawa Scale

CODING MANUAL FOR CASE-CONTROL STUDIES

***SELECTION (total= 4 points)***

1. **Case Definition Adequacy**
2. Preeclampsia (PE) defined as SBP/DBP>140/90 mm of Hg and proteinuria >300mg/24 hour or protein: creatine >0.3 mg, or urine albumin 2+ on the dipstick (ACOG)---1 point
3. PE criteria other than ACOG; biochemical parameters mentioned----1 point
4. PE criteria other than ACOG; biochemical parameters not mentioned----0 point
5. No description of PE criteria---0 point
6. **Representativeness of the Cases**
7. All eligible PE cases had sHLA-G done with trimester specification---1 point
8. No trimester specification---0 point
9. **Selection of Controls**
10. Normal pregnancies---1 point
11. Not specified on the control population---0 point
12. **Definition of Controls**
    1. Subjects not meeting PE criteria---1 point
    2. Subjects with other complications other than PE---0 point

# COMPARABILITY (total= 2 points)

1. **Comparability of Cases and Controls Based on the Design and/or Analysis.**
2. Availability of sHLA-G values of the same trimesters in PE cases and controls---1 point
3. Statistically compared with cases in terms of age, gestational age at sampling, and/or gravidity.

p<0.05---1 point

p> 0.05---0 point

Not compared statistically---0 point

# EXPOSURE (total= 4 points)

1. **Ascertainment of Exposure**

PE cases retrieved from secured records---1 point

PE cases identified from clinical criteria and diagnostic testing---1 point

No description---0 point

1. **Same method to ascertain exposure in cases and controls**

Yes---1 point

No---0 point

1. **Non-response rate**

Same rate for both groups---1 point

Rate different between groups---0 point

**Supplementary table:** Risk of bias scores of the included studies as assessed using NOS for case-control studies.

| **Study** | **Selection** | | | | **Comparability** | **Exposure** | | | **Total score** |
| --- | --- | --- | --- | --- | --- | --- | --- | --- | --- |
|  | Case definition adequacy | Representativeness of the cases | Selection of controls | Definition of controls | Comparability between cases and controls | Ascertainment of exposure | Same method of ascertainment | Non-response rate |  |
| Biyik et al. 2014 | 1 | 1 | 1 | 1 | 2 | 1 | 1 | 1 | 9 |
| Garcia et al. 2018 | 1 | 1 | 1 | 1 | 1 | 1 | 1 | 1 | 8 |
| Jacobsen et al. 2020 | 1 | 1 | 1 | 1 | 1 | 1 | 1 | 1 | 8 |
| Kolarz et al. 2012 | 1 | 1 | 1 | 1 | 2 | 1 | 1 | 1 | 9 |
| Marozio et al. 2017 | 1 | 1 | 1 | 1 | 1 | 1 | 1 | 0 | 7 |
| Rokhafrooz et al. 2018 | 1 | 1 | 1 | 1 | 2 | 1 | 1 | 1 | 9 |
| Steinborn et al. 2003 | 1 | 1 | 1 | 1 | 1 | 1 | 1 | 0 | 7 |
| Steinborn et al. 2007 | 1 | 1 | 1 | 1 | 1 | 1 | 0 | 0 | 6 |
| Yie et al. 2005 | 1 | 1 | 1 | 1 | 1 | 1 | 1 | 1 | 8 |

**Data availability statement**

All required data is in the manuscript itself.
